# Asthma—snapshot or motion picture?

**DOI:** 10.3389/fgene.2013.00073

**Published:** 2013-04-30

**Authors:** Anabela G. Berenguer, Alexandra Rosa, António Brehm

**Affiliations:** ^1^Human Genetics Laboratory, University of MadeiraFunchal, Portugal; ^2^Medical Sciences Unit, Life Sciences Center, University of MadeiraFunchal, Portugal

Asthma is a complex disease physiologically characterized by shortness of breath, coughing, and wheezing (Holgate, 2011). In response to a variety of stimuli, the airways become more sensitive leading to bronchial hyperresponsiveness (Sterk and Bel, 1989; Scichilone et al., 2006; Kang et al., 2012). Consequently, in a process known as bronchoconstriction, airways become narrower, impeding the normal airflow into and out of the lungs (WHO, 2011), by contraction of the bronchial smooth muscle (EPR-3, 2007). In addition, increased production of mucus occurs, further contributing to airway obstruction (EPR-3, 2007). Asthma is a chronic inflammatory disease, which if untreated can lead to structural changes in the smooth muscle and may result in airway remodeling (EPR-3, 2007).

At the molecular level, asthma usually involves a T-helper 2-type cell response (Th2)-(Lloyd and Hessel, 2010; Wenzel, 2012). The antigen-presenting cells (APC), including dendritic cells, present the antigen to the T-cell precursor, through the major histocompatibility complex II (MHCII) molecule coupled to the T-cell receptor (Kim et al., 2010). This leads to the activation of Th2 pathway, with the production of IL4, IL5, and IL13 cytokines and the consequent activation of B-lymphocytes and production of plasma cells, these last responsible for IgE production (Wenzel, 2012). Subsequently, basophils, eosinophils, and mast cells are activated, amplifying the allergic inflammation (Lloyd and Hessel, 2010; Wenzel, 2012).

More than 100 genes have been implied in asthma susceptibility across populations (Bijanzadeh et al., 2011; Torgerson et al., 2012; Zhang et al., 2013) while the association of environmental factors ranges from excessive cleanliness, constituting the “hygiene hypothesis” (Graham-Rowe, 2011), to poor socioeconomic development (Neto et al., 2012) and smoke exposure (Burke et al., 2012) to “anything and asthma” (Buchanan et al., 2006). Furthermore, replication studies of asthma candidate genes are often inconsistent (Rogers et al., 2009). Among the main reasons pointed out for this lack of replication are the populations' heterogeneous genetic backgrounds and their interaction with environmental factors, the different study designs and the lack of statistical power of the studied sample sets (Cardon and Bell, 2001; Nicolae and Ober, 2009; Grant and Hakonarson, 2010). Additionally, it is believed that in complex diseases many factors with weak effect rather than few with strong predictive power are thought to contribute to the disease susceptibility (Buchanan et al., 2006; von Mutius, 2009).

However, and despite of our current understanding of the biology and the contribution of environmental and genetic factors (Vercelli, 2008; Mukherjee and Zhang, 2011; Antó, 2012; Kumari and Rana, 2012), asthma is still a puzzling concept. The identification of causal factors and their contribution to complex diseases remain mostly unanswered questions, given the lack of robustness, inadequacy and/or limitations of many of the present-day methodologies (Buchanan et al., 2006). In addition, the broadly used case-control and GWA approaches, designed to unveil genetic variants underlying multifactorial diseases, do not take into consideration the evolutionary history of each biological trait (Buchanan et al., 2009).

The increasing incidence of complex diseases in the human populations suggests a high frequency of deleterious genetic variants (Kryukov et al., 2007). One may speculate that these genetic variations could have been beneficial or neutral in the past but have become detrimental as a result of changes in the surrounding environment and lifestyle of contemporary societies (Kryukov et al., 2007). A classical example supporting this idea is the “thrifty genes hypothesis” (Neel, 1962), sustaining that genotypes that once were protective against food scarcity are currently predisposing to obesity and diabetes, due to the current abundance in food resources and sedentary lifestyle (McDermott, 2006; Kryukov et al., 2007; Vardi and Bloch, 2008). Given the interface between genes and environment underlying this premise, it has been proposed that not only genetic but also epigenetic factors might be involved in the heritability of type 2 diabetes (Goh and Sum, 2010; Pijl, 2011).

Epigenetics, the study of changes in DNA expression that do not imply changes in the DNA sequence (Miller and Ho, 2008) but can be transgenerationally transmitted (Anway et al., 2005), have been transforming the way complex diseases and their risk are perceived (Miller and Ho, 2008; Feinberg and Irizarry, 2010; Relton and Smith, 2010). There is increasing evidence that epigenetic patterns can be altered by environmental factors since as early as *in utero* life (Fraga et al., 2005; Relton and Smith, 2010; Thornburg et al., 2010; Durham et al., 2011). Ethnic differences in human DNA methylation have been observed between an African and an European population (Fraser et al., 2012) and methylation-associated SNPs (mSNPs) were also found to exist, in which one of the alleles was associated with higher levels of methylation (Fraser et al., 2012). Furthermore, given a particular environmental exposure, genetic variants affecting the susceptibility to DNA methylation (*meth*QTL-methylation quantitative trait loci) can modify the response of modifiable genetic variants (*mod*GV), influencing the expression of the disease phenotype. This finding may partially explain conflicting results among several genetic studies (Karmaus et al., 2013). Taken together, these findings prompt us to a combined action of environment and genetics to epigenetic signatures, therefore modulating the genetic basis of a trait or disease.

While many genes are potential targets for epigenetic modification we will mainly focus our remarks on *IL4*. IL4 is a key cytokine involved in inducing IgE production via Th2 pathway (Oh et al., 2010), central in allergic response (Minton, 2008). *IL4* DNA methylation appears to be important in T-helper cell differentiation: while methylation of a highly conserved region at the 3′ end of *IL4* gene drives Th1 differentiation, demethylation of sites within the first intron of *IL4* results in enhanced IL4 production and Th2 differentiation (Miller and Ho, 2008) which is associated with an atopic asthmatic phenotype. Further, evidence of the importance of IL4 comes from observations of individuals with atopic asthma. Asthmatics who were sensitized to house dust mite extract (*Dermatophagoides pteronyssinus/Dermatophagoides farinae*) demonstrate a decreased level of methylation in the *IL4* gene that was strongly correlated to their IL4 concentration (Kwon et al., 2008).

The *IL4-c.590* C/T SNP variant (rs2243250) is located in the *IL4* promoter region, with allele *IL*4-590^*^T being associated to a 3-fold increase in *IL4* transcription and expression, given its extra NFAT transcription activator binding site (Rockman et al., 2003) and/or likely association of the SNP to methylation patterns, to our knowledge yet to be tested.

The frequency of *IL*4-590^*^T varies across populations, from 10.5% in Madeira Island population (Berenguer et al., 2012) to 11.3% in the South of England (Howell, 2004), and 15% in Spain (Leon et al., 2006). However, when considering Africans, the allele frequency increases dramatically, reaching 54.4% (Burchard et al., 1999), 59.2% in Cabo–Verde, and 76.5% in Guinea–Bissau (Berenguer et al., 2012). This frequency variation across present-day populations was likely shaped by positive selection, particularly influenced by pathogens (Fumagalli et al., 2009; Casto and Feldman, 2011). The *IL*4-590^*^T allele has been in fact associated with elevated anti-malarial IgG and IgE levels (Luoni et al., 2001; Farouk et al., 2005) and inversely correlated with parasitaemia in different African populations (Tangteerawatana et al., 2007). Because the Th2 response—in which IL4 plays a key role (Fumagalli et al., 2009)—is likely to be advantageous in tropical regions, where the likelihood of helminthic infection is higher (Le Souëf et al., 2006), differences in the allele frequencies across populations are likely to have occurred by natural selection (Le Souëf et al., 2006). The same allele has been linked to asthma susceptibility in a number of studies, mostly in Eurasian populations (Wang et al., 2004; Gervaziev et al., 2006; Kabesch et al., 2006; Chiang et al., 2007; Li et al., 2008; de Guia and Ramos, 2010; Berenguer et al., 2012) likely a side-effect of *IL4* positive selection, acting not only over genetic and environmental factors, but also over epigenetic signatures shaped through time.

One of many questions concerning asthma that have been in discussion over the last 20 years is the apparent sudden increase of asthma prevalence in westernized societies (von Mutius, 1998; Bloomfield et al., 2006; Umetsu and DeKruyff, 2006; Graham-Rowe, 2011) and, in particular, the implications on the disease incidence in developing countries, where selection has favored the Th2–cell immune response (Le Souëf et al., 2006). On one hand, the “hygiene hypothesis” postulates that increased hygiene and reduced exposure to microbes could be responsible for the increase in asthma prevalence in developed countries (Romagnani, 2007; Brooks et al., 2013). On the other hand, exposure to outdoor pollution resulting from traffic and other sources has been also associated to asthma in a number of studies (Lee et al., 2006; McConnell et al., 2010; Patel et al., 2011; Tzivian, 2011). It has been recently shown that methylation levels in the promoter of Neuropeptide S Receptor 1 (*NPSR1*) gene were associated to asthma in both children and adults as a result of smoking exposure (Reinius et al., 2013). Another recent study suggests that promoter variants in *NOS2* encoding for the inducible nitric oxide synthase (iNOS), when exposed to particulate matter with aerodynamic diameter ≤2.5 μm (PM_2.5_), were found to influence iNOS methylation pattern and thus affect the concentration of nitric oxide in exhaled breath (FeNO) levels, FeNO being considered a predictor for the future risk of asthma and wheeze (Salam et al., 2012). Interestingly, simultaneous exposure to inhaled diesel particles and allergen were found to induce hypomethylation within a CpG^−408^ site of the *IL4* gene promoter *in vivo* correlating to IgE production putting forward a new model for the aetiology of asthma (Liu et al., 2008).

These observations provide evidence that the role of the environment on epigenetic “angels and devils over DNA” should be of central interest to research.

Although, asthma is a likely intricate network of factors and these observations regarding *IL4* gene cannot be considered *per se*, they can still provide the opportunity for reflection about the current understanding of asthma targeting for new strategies, aimed to understand complex multifactorial diseases.

Rather than a present-day snapshot, asthma is more likely a motion picture across evolutionary time, in permanent interaction with the surrounding environment.
